# The visual system does not operate like a camera

**DOI:** 10.1167/jov.25.3.2

**Published:** 2025-03-04

**Authors:** Michele Rucci, Ehud Ahissar, David C. Burr, Igor Kagan, Martina Poletti, Jonathan D. Victor

**Affiliations:** 1 Department of Brain and Cognitive Sciences, University of Rochester, Rochester, NY, USA; 2 Center for Visual Science, University of Rochester, Rochester, NY, USA; 3 Department of Brain Sciences, Weizmann Institute of Science, Rehovot, Israel; 4 University of Florence, Italy; 5 Institute of Neuroscience, National Research Council, Pisa, Italy; 6 Decision and Awareness Group, Cognitive Neuroscience Laboratory, German Primate Center, Goettingen, Germany; 7 Feil Family Brain and Mind Research Institute, Weill Cornell Medical College, New York, NY, USA

**Keywords:** spatial vision, temporal vision, eye movements, visual dynamics, neural encoding

## Introduction

We welcome the editors’ invitation to reply to Dr. Gur’s Perspective (Gur, 2024), in which he argues that visual perception is entirely driven by snapshot images resulting from saccade landings and against the idea that vision is a dynamic process that incorporates information present in the temporal transients delivered by all kinds of eye movements. In Gur’s Opinion, saccade landings act as image flashes and elicit neural responses that dominate the entire periods of fixation, so that, contrary to recent findings, the small eye movements that continually occur during fixation serve no perceptual function.

Unfortunately, Gur’s Perspective is affected by numerous flaws, including the neglect of a large body of literature, misconceptions concerning proposed dynamic theories, inaccurate portrayals of eye movements and neural responses, arbitrary and unjustified assumptions on neural processing, erroneous interpretations, and multiple factual errors concerning previous experimental findings and procedures. Practically every point raised against dynamic theories, and almost every point raised in favor of saccade landing acting as an instantaneous flash, contains inaccuracies and/or misplaced assumptions. Once these errors are recognized, Gur’s proposal becomes baseless and illogical.

This letter aims to clarify the major misconceptions present in Gur’s Perspective and correct the most glaring inaccuracies. In Gur’s defense, we acknowledge that his Perspective follows the traditional camera-like view of the visual system, the pervasive view based on explicit spatial processing and representations that match naïve introspection. Because many readers may only be familiar with this textbook view, we briefly summarize the concepts of dynamical vision before addressing Gur’s Perspective.

Recognizing that vision relies on the dynamic signals provided by all kinds of eye (and body) movements opens up important new areas for experimental and theoretical studies, including the extent to which eye movements can be tuned and controlled to improve performance, how these control signals are naturally generated, and how planning, oculomotor, and visual signals interact.

### Vision as a dynamic process

**Figure 1.**
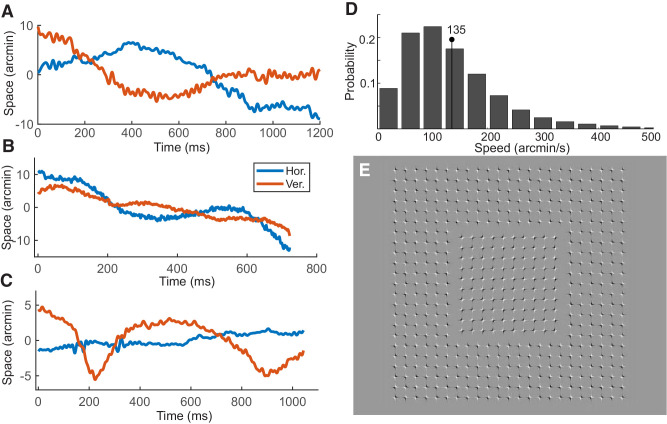
Inter-saccadic eye drifts. The eye moves considerably during natural fixation. Examples of real (no drawings) eye drift traces acquired with three instruments: (**A**) a digital dual Purkinje Image eye-tracker (Wu et al., 2023); (**B**) an adaptive optics scanning laser ophthalmoscope (Moon et al., 2024; Roorda et al., 2002); (**C**) the oscillating field monitor, a specially designed coil-based system that enables recording of fine eye movements without restraining the head (Eibenberger, Eibenberger, & Rucci, 2016). (**D**) instantaneous speed distribution of eye drift during normal head-free fixation (from Aytekin, Victor, and Rucci (2014)). These data were measured with the Maryland revolving field monitor, another custom instrument developed for high-resolution head-free eye tracking (Steinman, Levinson, Collewijn, & der Steen, 1985). (**E**) An image that makes the fixational motion of the eye apparent. Perceived motion occurs irrespective of whether the image is displayed on a pulsating CRT monitor, non-strobe LCD/OLED monitor, or printed on paper, directly showing that fixational eye movements elicit neural responses that do not depend on CRT frame-by-frame flashes (Image by Akiyoshi Kitaoka).

In the mid-20th century, careful recordings confirmed that the human eyes are never still—they continue to move even when we try to maintain steady gaze on a point. These movements are far from negligible, shifting stimuli quite rapidly across many receptors, especially in the fovea, where cones are most densely packed (Figure 1). Importantly, these movements do not reflect unavoidable noise (Barlow, 1952), and it was soon observed that they represent a form of slow control (Steinman, Haddad, Skavenski, & Wyman, 1973). Additionally, it was discovered that vision tends to fade away when stimuli are immobilized on the retina (Steinman & Levinson, 1990) and that retinal neurons are most strongly sensitive to temporal luminance changes (Lee, 1996). These observations are at the foundation of the so-called dynamic theories of vision, which argue that the perception of spatial relationships relies on luminance changes induced by both eye movements and environmental changes, all encoded by a moving retina (Ahissar & Arieli, 2001; Rucci, Ahissar, & Burr, 2018; Rucci & Victor, 2015). In this view, information about space is encoded in the form of a spatiotemporal code. This means that the temporal structure of activation of receptors is as important as their spatial location.

Although the first proposals that vision relies on changes rather than stationary images date back over a century with the dynamic theories of visual acuity (Arend, 1973; Averill & Weymouth, 1925; Marshall & Talbot, 1942), these theories lost traction in later decades when they seemed to be contradicted by experiments that attempted to eliminate retinal image motion (Riggs, Ratliff, Cornsweet, & Cornsweet, 1953; Tulunay-Keesey & Jones, 1976). In parallel, the rise of a reductionist approach in vision research aimed at elucidating spatial processing led to a focus on static conditions dominated by studies in anaesthetized animals and unnatural conditions of sustained fixation in humans. Unfortunately, during this shift, the previous insights were forgotten, and vision came to be hypothesized as based solely on spatial coding—the so-called camera model.

This model, which relies on a hierarchy of spatial processing operations, has become the standard textbook model despite the conceptual difficulties and the implausible assumptions it entails. Gur’s Perspective provides an excellent example, with its assumptions that the eye’s landing after a saccade creates a flash-like imprint of the image on the retina, allowing for spatial-only decoding of image details, akin to a camera. But saccades are not instantaneous, the stimulus moves over the retina both before and after saccade landing, and more so in the presence of normal head and body movements, there is no “shutter” in the visual system to freeze the image, and retinal integration times are on the order of tens of milliseconds. In other words, during natural viewing, there is no moment in which the visual system experiences “a frozen input,” as in the case of simplified computer simulations.

A critical observation that was ignored both in the 1970s and in Gur’s Perspective is that the pioneering experiments that supposedly contradicted dynamic theories suffered from serious technological and methodological limitations (Kelly, 1979). In the last 20 years or so, accurate measurements of eye movements, carefully tailored experiments with humans, neurophysiological results in macaques and humans, and computational analyses, as well as comparisons with other dynamical sensory modalities, have revived and expanded the dynamic theory of vision. This theory takes on different forms, but its core concept remains the same: visual encoding is a spatiotemporal process and eye movements play a crucial role to this process.

### Correcting misconceptions about active space–time encoding

At the core of Gur’s Perspective are fundamental misconceptions about proposed theories of dynamic vision. Although Gur lumps together proposals from separate groups, his criticism is directed toward the active space–time encoding theory, i.e., the idea that spatial encoding makes use of visual input transients resulting from eye movements (Ahissar & Arieli, 2001; Rucci, Ahissar, & Burr, 2018). There are several areas of confusion.

### Transients matter

A major and pervasive misconception in Gur’s Perspective concerns the proposed mechanisms of visual encoding.

The space–time encoding idea argues that temporal changes in the input signals—not fixational eye movements per se, as Gur seems to have understood—are necessary for visual perception. During natural viewing, temporal transients on the retina come from various sources, including moving objects in the scene, changes in illumination, and the motor activity of the observer. According to active space–time encoding, both externally and internally generated transients contribute to visual representations depending on the specific spatial information that they make available within the temporal bandwidth of sensitivity of the visual system (at non-zero temporal frequencies). Under natural viewing conditions, the most common types of transients come, by far, from the observer’s motor behavior, eye movements in particular, with their continual alternation between fast saccades and slow drifts. Consequently, studies on active space–time encoding have focused on the consequences of various types of eye movements and their interplay.

In specific conditions, like laboratory environments, other transients may be elicited. For example, stimuli may suddenly appear or disappear on the display, something that occurs in modern man-made interfaces but rarely happens in the natural world in which vision evolved. According to active space–time encoding, the temporal structure by which spatial information is delivered—that is, the sequence of stimuli on the retina—is critically important. Thus, one needs to be careful in extrapolating results obtained with artificial transients to natural viewing. Gur’s Perspective misses this fundamental concept and completely disregards the dynamics of visual stimulation in the literature and demos it cites. In fact, if one pays attention to the temporal format of stimulus presentation, it becomes evident that the literature cited by Gur as evidence against the space–time encoding idea is actually fully compatible with it.

### Flashed stimuli are powerful, but unnatural, transients

To provide a more specific example of this general issue, consider the case of stimulus flashes. Gur argues that space–time encoding is “incompatible with our faithful perception of briefly displayed objects.” In his view, because substantial information can be extracted from a brief flash over a blank screen, ocular drift-related motion cannot be useful. Leaving aside concerns of logical rigor, this criticism is unfounded: the space–time encoding proposal actually predicts that isolated flashes are highly effective stimuli. This is because a flash preceded and followed by a uniform field creates an approximately equal replica of the spatial spectrum of the flashed image at any nonzero temporal frequency. In other words, flashes deliver uniquely powerful transients that convey full information about the spatial structure of the stimulus.

There are several points, however, that need to be considered further. Brief isolated flashes very rarely occur during natural viewing and their luminance transients differ profoundly from those delivered by eye movements, including saccades. Unlike flashes, the luminance transients from eye movements produce a major reformatting of spatial information, emphasizing selected bands of spatial frequencies according to the velocity characteristics of eye motion (Mostofi et al., 2020). Furthermore, in contrast with tachistoscopic laboratory conditions, the visual scene is always present during natural viewing. This implies that a) the normal alternation between various types of eye movements continually structures the luminance flow impinging onto the retina, and b) the visual system can integrate the spatial information delivered by different types of transients. Thus, one has to be extremely careful in extrapolating results obtained with isolated transients—like a flash—to natural viewing.

Indeed, previous work has shown that, during the normal saccade–drift alternation, the dynamics of visual processing follows the evolving power distributions of the input transients resulting from eye movements. High spatial frequency information is integrated across saccades and drifts, whereas low spatial frequency information is derived primarily from saccade transients, yielding coarse-to-fine dynamics at every fixation (Boi, Poletti, Victor, & Rucci, 2017). Similar considerations apply to the luminance transients produced by eye blinks, which are similar across all spatial frequencies and deliver stronger signals than saccades in a low-frequency band. During normal active fixation, while the eye drift modulations enhance sensitivity to high spatial frequencies, blink transients primarily enhance perception at low spatial frequencies, improving visibility of the coarse, low-resolution structure of the visual scene (Yang, Intoy, & Rucci, 2024).

In sum, from the Perspective of space–time encoding, brief flashes are such powerful stimuli because they generate transients exceptionally rich in spatial information. These transients, however, engage temporal encoding mechanisms typically activated by other types of dynamic changes, namely, those resulting from the observer’s motor behavior. Importantly, because the occurrence and characteristics of behaviorally induced transients can be controlled by the observer, the visual system has the flexibility to continually adjust the combination of blinks, saccades and drifts according to ongoing demands, tuning visual representations to the task at hand.

### All eye movements contribute

Another area of misconception regards the role of different types of eye movements. Gur writes that space–time encoding “is incompatible with physiological data showing that all information is conveyed by the short neural volleys generated when the eyes land on a target.” However, he does not seem to realize that a) there are no existing data showing that all information is conveyed by such volleys, and b) according to the space–time encoding proposal, the modulations from all eye movements, and saccades (including fixational saccades, or “microsaccades”), as well as fixational drifts, contribute useful spatial information as afforded by their characteristics (Rucci, Ahissar, & Burr, 2018).

**Figure 2.**
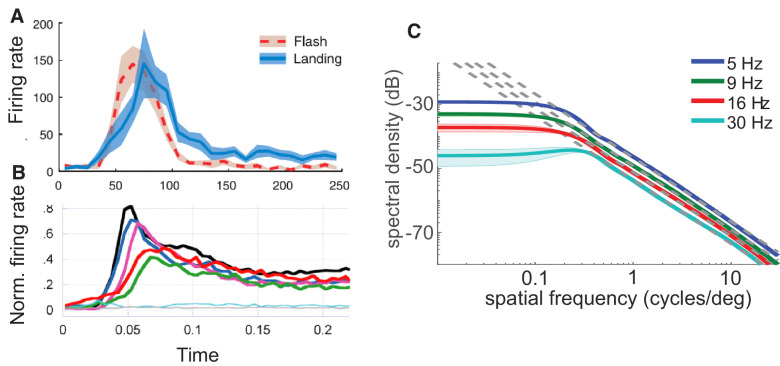
Comparing saccades to flashes. (**A** and **B**) The data cited as source for Gur’s hand drawings do not support his claims. (**A**) Data from Kagan, Gur, and Snodderly (2008). V1 responses to stimuli that were either flashed and immobilized on the retina or brought in the receptive field by a saccade over a uniform field and then moved normally because of fixational drift. Responses to flashes and saccades differ even in this simplified scenario in which lack of a background led to a more similar stimulation in the two conditions. Note that even transient cells (the focus of this analysis in Kagan et al.) exhibit more sustained responses during the drift period, beginning at approximately 100 ms. Also compare the very rapid response decay with Gur’s drawing (time zero marks saccade or flash onset). (**B**) Data from figure 6 in (Ruiz & Paradiso, 2012), the data that most closely resemble Gur’s sketches. Each curve represents a specific experimental condition. Gur arbitrarily chose the two curves (the black and the blue) that are closest to each other. But his text compares the natural responses elicited by saccades over a scene (which is the green curve) to a flash of the scene, a condition that was not present in Ruiz and Paradiso’s study. (**C**) Input signals from saccade transients and flashes differ. Because of their kinematics, saccades deliver luminance transients that equalize (whiten) the power of natural scenes in an amplitude-dependent low spatial frequency range. Solid lines show the power delivered by saccades of 2° to 3° amplitude at several temporal frequencies during the viewing of natural scenes. Dashed lines are proportional to the power delivered by flashes of the same scene. Note the departure at low spatial frequencies (from Mostofi et al., 2020).

Active space–time encoding does not make a sweeping claim that fixational drifts are the only ocular movements that are “essential for good visibility” (as stated by Gur), but rather that they add information specifically in a range of high spatial frequencies. Accordingly, the theory does not claim that saccade landings are irrelevant for visual perception as Gur’s Perspective implies, but rather that saccades contribute powerful visual transients that convey spatial information over a wider frequency band than that covered by fixational drift, extending sensitivity to lower spatial frequencies than drift (Boi et al., 2017; Mostofi et al., 2020). Critically, the luminance transients delivered by saccades differ drastically from those of flashes or contrast steps, as explained in detail later in this letter (see Figure 2C).

Thus, the space–time encoding theory proposes that saccades and fixational drifts, together, provide information across a broad range of spatial frequencies, based on the specific way each movement reformats spatial patterns into spatiotemporal input signals. In line with this proposal, studies in macaque V1 (co-authored by Gur) elucidated distinct neuronal classes: those that respond transiently to saccades, those that respond continuously during drifts, and a combination of both (Kagan et al., 2008; Snodderly, Kagan, & Gur, 2001).

### Efficient encoding and no need for explicit space representation

Contrary to Gur’s assertion, active space–time encoding also leads to efficient representations. The visual input signals reformatted by eye movements discard redundant information in natural scenes before any neural processing (Kuang, Poletti, Victor, & Rucci, 2012; Mostofi et al., 2020), enabling a compact and metabolically less demanding transmission of visual information from the retina to the cortex. These advantages do not come at the expense of rapid processing, because the spatiotemporal input signal leads to synchronous firing in the retinal output when a contour is crossed. Furthermore, unlike the camera-like model, which unrealistically requires all spatial information to be simultaneously transmitted to the cortex through the limited-capacity channel of the optic nerve, active space–time encoding enables task-dependent control of the flow of information, keeping inter-saccadic intervals short in tasks that can primarily rely on low spatial frequencies and prolonging fixation when high-acuity vision is needed.

Gur also argues that space–time encoding presents the additional difficulty, “that somewhere, somehow, this code must be decoded into a parallel spatial one when reaching perception.” However, this statement seems based on a homunculus view of perception, one that needs explicit reconstruction of the spatial image in the brain. There is no need for explicitly decoding spatial information; it is not lost. Spatial information is just present in a different format than a purely spatial image, encoded in the spatiotemporal flow of neuronal activity and oculomotor dynamics.

### Inaccuracies and errors in Gur’s Perspective

The rest of this letter is dedicated to address in detail the major inaccuracies at the foundation of Gur’s Perspective.

### Inaccurate characterization of eye movements and saccade-fixation dynamics

Much of Gur’s argument relies on an inaccurate assumption about the characteristics of eye movements and the saccade-fixation dynamics. This is evident in his figure 1, which, rather than showing real eye movement traces, is a hand-drawing. Note that to support Gur’s point that saccade act as “flashes,” the sketch depicts saccades as lasting only a few milliseconds. But saccades are not instantaneous; they move from one point of fixation to the next with a very specific dynamics that lasts tens of milliseconds. For a broad range of visually relevant spatial frequencies, these dynamics generate transient signals that are well-matched to the sensitivity of retinal ganglion cells (Mostofi et al., 2020).

Figure 1 in Gur’s Perspective also reveals another misconception. As illustrated by the very brief inter-saccadic interval in his drawing, Gur claims that saccades occur so frequently (“∼4 times a second”) that there is no time left for fixational eye movements to elicit a meaningful response. But, inter-saccadic intervals typically exhibit broad distributions (Guy, Lancry-Dayan, & Pertzov, 2020; Otero-Millan, Troncoso, Macknik, Serrano-Pedraza, & Martinez-Conde, 2008) and, as explained elsewhere in this letter, there is plenty of time for inter-saccadic visual signals to be generated and used even during brief fixations. Moreover, saccades occur much less frequently in tasks that require fine examination of the visual scene, i.e., those in which the modulations from fixational drifts are particularly helpful. For example, fixations tend to reach a second or more when reading an eye chart (e.g., Intoy & Rucci, 2020) or when performing other high-acuity tasks (e.g., Intoy, Mostofi, & Rucci, 2021; Shelchkova, Tang, & Poletti, 2019). Long and variable drift periods have also been reported in fixating monkeys (e.g., an average fixation duration of 770 ms in Kagan et al., 2008, see their Supplementary Figure S14).

Gur’s Perspective also grossly misrepresents the characteristics of fixational drift, the incessant motion occurring in between saccades. Eyeballing data from Ratnam, Domdei, Harmening, and Roorda (2017), which mentions that in their experiments “the stimulus traversed a retinal distance equaling about 10.5 unique cones during each 750-ms,” Gur concludes that the speed of ocular drift is just 10’/s. But this estimate is affected by several flaws. First, Gur missed that the stimulus was not presented at the very center of gaze, where cones are the smallest. Second, this statement enables estimation of the span of motion, not the speed: ocular drift resembles Brownian motion, and to cover this average span, instantaneous speeds must be much faster. Third, speed measurements depend on several factors, including a) the task (Intoy & Rucci, 2020; Lin, Intoy, Clark, Rucci, & Victor, 2023), b) how the measurements are acquired, c) how the trials are selected, and d) how the data are processed. Lack of consideration of these factors leads to inaccurate generalizations. In data recorded with dual Purkinje Image eye-trackers and eye coils, filtering around 30 Hz, gives average speed in high-acuity tasks of 40-50′/s (see Figure 1), and comparable speeds have been reported in fixating monkeys in studies that Gur co-authored (e.g., Kagan et al., 2008; Snodderly et al. 2001). Fourth, one needs to consider that most measurements are obtained with the head immobilized. These measurements underestimate the retinal motion that occurs under natural viewing conditions in which the head is free to move: under these circumstances, average fixation speeds are higher than 1 deg/s, as shown in Figure 1D (Aytekin et al., 2014; Steinman et al., 1985).

### Incorrect estimation of time for drift-induced neural responses

Another misconception regards the presumed lack of time for neurons to respond to drifts. Gur states “Given that the strong transient neural volley resulting from the landing saccade lasts at least 80 msec into the pause before starting to moderate (see figure 2), and that preparation for the next saccade starts 100 msec before the end of the fixational pause (Rolfs & Carrasco, 2012), there are only 70 msec, in a 250 msec pause, where drift may be effective [...] Now, it takes a 2’ drift to enable a 1’ RF to fully cross a 1’ spatial element. At 10’/sec drift velocity (cf., figure 2, Ratnam et al., 2017), a 2’ drift lasts 200 msec which is much longer than the 70 msec ‘effective’ drift window.”

This conclusion relies on multiple incorrect assumptions. As explained elsewhere in this letter, a) fixation intervals are much longer in high-acuity tasks, those in which modulations from drift are useful, and b) drifts move the eyes much more rapidly than Gur believes, which would allow for strong responses even in brief intervals. However, there are other layers of misconception as well. First, Gur assumes that the visual system cannot make use of retinal responses elicited by fixational drift during saccade preparation. Presumably, this is because he believes in strictly serial processing between perception and action in which all relevant information from the current location must be gathered before the next saccade is planned. This assertion goes against a large body of evidence demonstrating parallel processing during the active vision cycle. Second, Gur assumes that the stimulus needs to move to a new cone on the retina to elicit a response. However, it is well-established that subtle movements, on the order of a few micrometers, can evoke vigorous discharges in ganglion cells (Nelson, 2007; Shapley & Victor, 1986).

Related to this point, Gur also claims that the “response to the landing saccade dominates the entire drift period,” therefore leaving little time for drift responses to exert an action. He concludes that “any slowly-accumulated weak responses that may be due to the drifting eye are negligible relative to the strong persistent volley generated by the landing saccade.” However, it is well-established that there is a wide diversity in the strength and the timing of neural responses (Snodderly, 2016): at the two ends of the continuum, “high-pass” neurons exhibit rapid transient responses to saccades and do not respond during drifts, while “low-pass” neurons start responding later and express sustained firing during entire drift periods (Kagan et al., 2008; Snodderly et al., 2001). Furthermore, unless the stimulus is perfectly immobilized on the retina after a saccade, it is not possible to distinguish the spikes elicited by ensuing ocular drift from those resulting from the preceding saccade. This is made very clear in Kagan et al. (2008), one of the main articles cited by Gur in support of his statement. Because of the difficulty in determining whether spikes were triggered by the mere presence of the stimulus within the receptive field or the retinal motion caused by drift, both Snodderly et al. and Kagan et al. labeled these sustained responses as “position/drift-activated.”

In sum, Gur’s conclusion that “there is simply no time for the drifting eye to produce any meaningful response for even the smallest spatial elements” is baseless and illogical. This conclusion is not only not supported by any real data or facts, it also would not stand even if one were to take Gur’s numbers at face value: 130 ms of presumed saccade response (the arbitrary interval reported in the Perspective) would leave more than one-half a second of fixational drift in high-acuity tasks, when drift is most needed.

### Inaccurate comparison of neural responses elicited by saccades and flashes

A main point of Gur’s Perspective, portrayed in his figure 2, is that saccades and “flashes” (now meant as contrast steps from blank screens) are similar, which in Gur’s Opinion, somehow, excludes the possibility that cortical neurons also respond to fixational movements. This argument makes little sense given the large diversity in individual neural responses and the consideration that, unless the stimulus is stabilized on the retina, both responses to saccades and contrast steps may be equally affected by the motion caused by fixational eye movements. Moreover, the many illusions of apparent jittery motion at fixation (e.g., Murakami & Cavanagh, 1998) would obviously not be perceivable if the visual system were not sensitive to the motion signals caused by fixational eye movements (see Figure 1E).

Still, it is worth spending a few more words on this issue to make two observations. The first observation is that, as for many statements in Gur’s Perspective, there is a disconnect between claims and empirical data. Specifically, the very literature cited by Gur does not support his claim that neural responses to saccade and flashes are identical. As is the case for Gur’s Figures 1 and 2 are also not real data but hand drawings supposedly inspired by neurophysiological recordings. Even if one tolerates artistic departures from the data, these curves are not what the author claims them to be.

The caption of the figure mentions as sources two articles. The first article is Kagan et al. (2008); but no curve in Kagan et al. resembles the sketches. Furthermore, by stabilizing stimuli on the retina following contrast steps and comparing them to non-stabilized post-saccadic responses, Kagan et al. actually showed important differences in the effects of the two types of stimulation (see their figure 9C, reprinted here in Figure 2A). While physiological data indicate that both landing saccades and stabilized contrast steps can yield similar magnitudes at their peak responses (figure 6A in Kagan et al.), the dynamics of neural activity differ considerably: even transient neurons exhibit a shifted and more sustained response following saccades, when the stimulus on the retina moves normally because of fixational drift. This happens even in the absence of a background (stimuli were presented over blank fields), which should contribute to make retinal stimulation in the two conditions more similar to each other. The reader may want to compare the real data from Kagan et al. (Figure 2A) with Gur’s hand drawing in which flashes are sketched to elicit more sustained responses than saccades.

The other cited reference is Ruiz and Paradiso (2012), and indeed Gur’s sketches resemble two of the curves shown in figure 6 of the original article (replotted in Figure 2B). However, these curves do not match Gur’s description. The black curve in Figure 2B represents the response to a flash of a bar over a gray field, whereas the blue curve represents the response when a saccade lands on the same stimulus after crossing a picture. Because the latter condition is essentially a flash after the saccade transient, it is not surprising that the responses are somewhat similar: in both cases the receptive field of the neuron experiences the sudden onset of an ideally oriented bar over a uniform field. Gur’s description, however, compares the response from saccades on visual scenes (the green curve of the original figure) to flashes of the same visual scenes, a condition that is not present in Ruiz and Paradiso. Thus, it is puzzling why Gur refers to these articles as sources of inspiration for his drawings.

The second observation on this issue is that, critically, saccades and flashes are very different in terms of the visual signals they deliver to the retina. A brief pulse (or a step) of an image transforms a spatial image into a spatiotemporal signal that preserves the image structure (i.e., its spatial frequency distribution) at every temporal frequency (dashed lines in Figure 2C). In contrast, the luminance modulations delivered by a saccade differ in amplitude across spatial frequencies (continuous lines in Figure 2C). Within the range of temporal sensitivity of retinal ganglion cells, saccades transients equalize (whiten) the spectral distribution of natural scenes up to a critical spatial frequency that depends on saccade amplitude. This equalization is a computational step that has long been argued to be beneficial for early neural encoding (Atick & Redlich, 1992; Barlow, 1961; Srinivasan, Laughlin, & Dubs, 1982) and does not occur with flashes.

In sum, because saccades move from one location to the next via specific dynamics, they deliver spatiotemporal stimuli that, within the temporal range of retinal sensitivity, differ from the spatial image itself. This spatiotemporal reformatting occurs *before* any neural processing and is present in the input signals experienced by neurons. A camera-like model of the visual system needs to somehow invert this transformation either by counteracting it via unknown neural computations or by assuming an instantaneous reset of neural responses at saccade onset, as in Gur’s Perspective.

### Misplaced assumptions on drift randomness and encoding consequences

Gur believes that drift characteristics prevent encoding of spatial information in temporal modulations. He writes: “The erratic nature of the drift trajectory makes any space-to-time code impossible. Direction reversal and loops are often observed [...] Furthermore, even in a single subject repeatedly fixating the same target, saccade landing locations and drift trajectories differ between trials. Clearly no consistent space-to-time coding and decoding can be had under such conditions.” Again, this assertion is made without any accompanying logical explanation as to why variability in eye movements should make use of temporal information impossible. There are both logical and experimental grounds on which the assertion is incorrect. Most fundamentally, spatial information is not lost, it is encoded in the spatiotemporal structure of visual stimulation. Even if drift trajectories were uncontrolled and unmonitored, they would still useful, and spatial information can be efficiently decoded with minimal assumptions (Anderson, Ratnam, Roorda, & Olshausen, 2020; Burak, Rokni, Meister, & Sompolinsky, 2010). While the fixational motion is likely to affect the spatiotemporal dynamics of neural activity in many ways (Ahissar & Arieli, 2012), spatial information is also present in the instantaneous pair-wise correlation between responses (Desbordes & Rucci, 2007), as neurons will tend to be synchronized when they simultaneously cross a contour (Greschner, Bongard, Rujan, & Ammermuller, 2002; Segal et al., 2015). There is, therefore, no need for complex decoding strategies for making use of this information. Moreover, drifts seem to be both controlled in a task-dependent manner (Intoy & Rucci, 2020; Lin et al., 2023; Malevich, Buonocore, & Hafed, 2020; Steinman et al., 1973) and monitored via extra-retinal signals that contribute to fine spatial judgments (Raghunandan, Frasier, Poonja, Roorda, & Stevenson, 2008; Zhao, Ahissar, Victor, & Rucci, 2023), and there is also evidence that direction reversals increase drift-based temporal information (Gruber, Ullman, & Ahissar, 2021; Rivkind, Ram, Assa, Kreiserman, & Ahissar, 2022).

### Variability in neural response prevents space–time encoding

**Figure 3.**
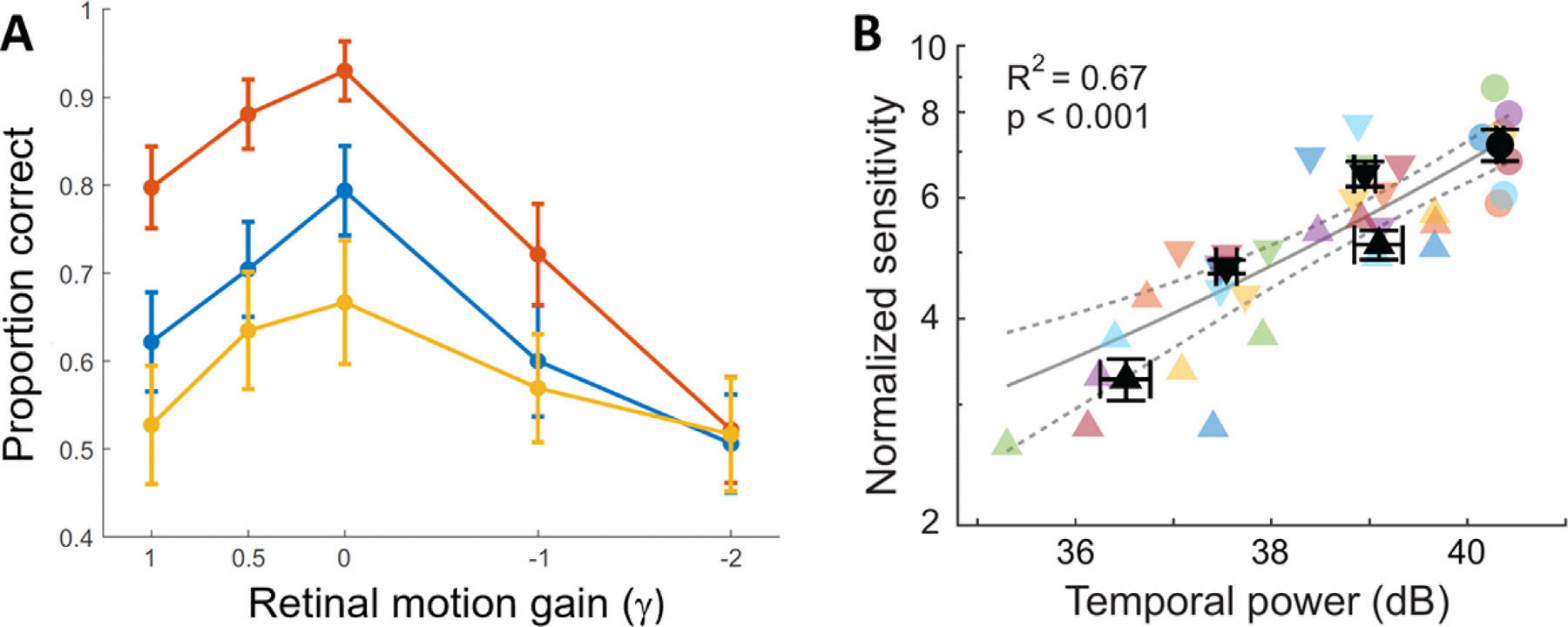
Performance in discriminating the orientation (±45°) of a 16 cycles/deg grating with controlled amount of retinal motion. (**A**) Results obtained with a non-strobing LCD display. A retinally-stabilized stimulus moved by means of a scaled version of a previously recorded fixational eye trace p(t). That is, following a change in eye position Δe(t), the stimulus moved on the display by Δs(t)=(1-γ)Δp(t)-Δe(t), where γ is the gain that controls the amount of retinal motion (γ = 0 normal motion; γ = 1 no retinal motion ). Each curve shows data from one subject. Errorbars are ± 1 SEM. (**B**) Performance in these experiments closely follows the strength of fixational luminance modulations, as predicted by active space–time encoding. Each symbol color is one subject; black symbols are averages across subjects. Adapted from Intoy et al. (2024).

Another unjustified assumption in Gur’s Perspective is that the variability in neural responses is too high to enable use of drift-induced responses (e.g., “Single cells response latencies and magnitude are quite variable. Gur and Snodderly (2006) showed that response variability was particularly high for low response rates, which is the case for the very few spikes that may be related to the drifting eye.”) However, a) population response latencies are likely to have a much smaller variability than single-neuron responses. And b), under anesthetized/paralyzed conditions, response timing changes of only 10 ms can be informative about contrast (see figure 3A of Reich, Mechler, & Victor, 2021). Furthermore, c) Gur’s claim neglects the fact that, to reach reliable conclusions about variability, one needs to accurately know where the stimulus is relative to the receptive field, which has long been a major challenge in neurophysiology (see Yates et al., 2024). Without accurate localization of gaze, it remains unknown how much of the neural variability actually results from changes in the spatiotemporal stimulus impinging onto the receptive field. d) There are several papers in the literature showing high precision in neuronal firing once fixational eye movements are included in the analysis. See, for example, figure 1 in Segal et al. (2015) or figure 2 in Greschner et al. (2002) for precise synchronization of neural responses during fixational eye movements. e) The recent study of Wu et al. (2024) addresses exactly the question of encoding precision in the primate retina, concluding that fixational eye movements enhance the precision of visual information.

### Misrepresentations of previous work

The Perspective reports inaccurate and false information about many previous findings in addition to those mentioned above (e.g., Kagan et al., 2008; Ruiz & Paradiso, 2012). Misrepresentations are particularly evident for previous studies examining the perceptual consequences of saccades. Gur writes: “*Two fairly recent studies (Boi et al., 2017; Mostofi et al., 2020), though, did consider the **perceptual effects** of the pre-fixation saccadic high velocity sweep and suggested that, say, 3–5° saccades shape the image such that at fixation start **very low SFs (<0.1 cycles/deg)** are enhanced. Consequently, fixational pauses can be divided **into two (unspecified) intervals; in the first, low SFs are enhanced, whereas during the later, drift dominated interval, high SFs are processed,** resulting in coarse-to-fine processing. However, such an approach is untenable*” (emphasis ours).

The sentence conflates Boi et al. (2017) and Mostofi et al. (2020). But these two studies are very different. Mostofi et al. (2020) do not deal with perceptual or neural responses at all. It is a power spectrum analysis of the visual input to the retina. That a saccade yields a stronger modulation than drift at low spatial frequencies is a matter of fact that primarily follows from saccades moving gaze further than drifts.

Boi et al. (2017) present a model-driven psychophysical study showing coarse-to-fine dynamics during post-saccade fixation. The model indicates that a cell with any given spatial sensitivity profile will shift its response toward higher spatial frequencies during the course of post-saccadic fixation because of the changes in its driving input (from saccade to drift). This effect leads to the prediction that vision relies primarily on saccade transients at low spatial frequencies and—contrary to Gur’s understanding—on the integration between saccade-induced and drift-induced modulations at high spatial frequencies, leading to coarse-to-fine dynamics. Psychophysical results show perceptual contributions from saccades and drift that are consistent with these modeling predictions. These results have already been replicated by other laboratories and used to improve the efficiency of virtual reality displays via gaze-contingent rendering (Kwak et al., 2024).

It is unclear why Gur believes that the saccade influence reported by Boi et al. (2017) is confined to very low spatial frequencies (Boi et al., 2017 used 1 cpd in their experiments, not <0.1 cpd as stated by Gur) or why the periods of saccade and drift influences are “unspecified” (figure 3E in Boi et al. (2017) shows a window of saccade influence of approximately 50 ms). More broadly it is unclear why Gur considers the approach “untenable,” as the main thrust of the work is that the post-saccade dynamics of human perception follows a coarse-to-fine dynamics of visual analysis consistent with many previous psychophysical and neurophysiological studies (Burr, 1981; Hegdé, 2008; Neri, 2011; Schyns & Oliva, 1994; Watt, 1987), a body of literature that is ignored in Gur’s Perspective.

Misrepresentations do not end here. Gur argues that the periods of stimulus exposure used in Boi et al. (2017) are too long (“in all cases, stimuli were presented for durations longer than those characterizing the saccade/drift cycle (cf., Boi et al., 2017, 800 and 2300 msec”). As mentioned, 800 ms is a perfectly reasonable fixation duration in high acuity tasks, and Gur missed that 2300 ms is a control condition to show that performance at low spatial frequencies is impaired without a saccade, even if one extends exposure to a very long time.

Furthermore, Gur has not understood the functioning of the model, as he seems to believe that the results in Boi et al. (2017) depend on inaccurate modeling of eye movements (“the authors’ analysis is based on the presumed continuity between high velocity saccades and the very low velocity drift; a continuity that ignores the intervening flash-like 1-2 msec deceleration that is saccade landing”). This is not the case. Boi et al. (2017) did not simulate eye movement: unlike Gur’s figures, Boi et al. (2017) was based on recording of real eye movements. Their conclusions did not incorporate nor depend on continuity between saccades and drift, which is irrelevant both in the model and perceptually. In fact, one gets a similar enhancement of low spatial frequencies also with eye blinks, a transient that more closely resembles a contrast step (Yang et al., 2024).

### Presumed lack of evidence for space–time encoding theories

As pointed out in the references cited in this letter, there is a large and growing body of evidence, ranging from human psychophysics to neurophysiology, supporting the notion that oculomotor transients provide useful spatial information. The interested reader is referred to Intoy et al. (2024), Yang et al. (2024), and Wu et al. (2024) for the most recent experimental validations of theoretical predictions.

However, Gur believes that there is no evidence supporting space–time encoding because “unfortunately, all studies, used either CRT monitors where each pixel is flashed with a sub-msec persistence time [...] images are never really drifting across the retina but rather are flashed many times on a ‘frozen’ retina [...] retinal stabilization of the pulsed display can potentially hamper visual performance through trivial mechanisms.” These statements are wrong at two fundamental levels. At an empirical level, retinal stabilization results obtained with CRTs have been replicated in non-strobe displays (Li & Rucci, 2024). See, for example, Figure S2 in Intoy et al. (2024) for a systematic manipulation of retinal stabilization with a non-strobe LCD. These data, reproduced in Figure 3, show that spatial sensitivity systematically varies with the amount of retinal image motion in proportion to the power of the induced luminance modulations, as predicted by active space–time encoding. This happens in the absence of a pulsating input, as luminance remains constant in between frames in this display. Similar results have also been obtained with OLED displays that ensure very steady stimulation in between frames (Wang & Rucci, 2024).

At a more conceptual level, Gur’s intuitive assumption that the stimulus is “frozen” on the retina and cannot be reformatted by eye movements when displayed via a train of brief flashes is also incorrect. Because of the displacement in the retinal image from one frame to the next, eye movements continue to redistribute power across temporal frequencies even during stroboscopic viewing, so that the input signal within the temporal bandwidth of visual sensitivity is actually very similar to that experienced with a stationary non-pulsating image. The reason for this is that the Fourier transform of a train of impulses is a stack of harmonics at integer multiples of the display frequency, including a component at 0 Hz. For each harmonic, eye movements will redistribute the power of the stimulus, exactly as they do with a stationary image, as the resulting input signal at every spatial frequency is given by the temporal-frequency convolution between the monitor output and the redistribution caused by eye movements (see Intoy et al. (2024) for details). Thus, even in an ideal stroboscopic display with infinitesimally brief pulses, the temporal power of retinal stimulation during fixational drift is more broadly distributed at high than low spatial frequencies, in the same way that it occurs for natural stimuli. It is also worth noting that CRT persistence is longer than what assumed by Gur, see figure 3B in Elze (2010) or figure 7 in Santini, Redner, Iovin, and Rucci (2007), which would further contribute to spread power across temporal frequencies.

Gur also goes back to one of the classical studies performed to test dynamic theories of visual acuity (“it is useful to look at a study (Keesey, 1960), where true stabilization was achieved by using a mirror attached to a contact lens”). Gur presents Keesey’s study as a case of true stabilization, even though there are many technical concerns with the stability provided by this classical approach (Kelly, 1979). Note that Keesey’s stimuli—which contained information over broad spatial frequency bands—were flashed over blank fields. Again, visibility of stimuli under such conditions is not only compatible, but predicted by, the proposal that information is encoded in the temporal domain. The pioneering experiments of last century faced many problems, ranging from the impossibility to selectively stabilize the image during periods of visual fixation between saccades to the studies’ lack of methods for objectively assessing the quality of stabilization. The interested reader is referred to Rucci, Iovin, Poletti, and Santini (2007), the first study that directly contradicted Keesey’s conclusions, for an overview of the various issues with the classical literature on retinal stabilization.

## Concluding remarks

We have attempted here to detail various fallacies and inaccuracies of Gur’s thesis, giving readers a scientific framework from which to draw their own conclusions. We firmly believe that vision is an intrinsically dynamic process, which can be fully understood only by considering its temporal and spatial properties concurrently. A stationary snapshot may be a convenient simplification to demonstrate, say, the optics of the eye, but does not begin to capture the reality of unconstrained and purposeful exploration of the world, through body, head and eye movements. And while we concentrate here on the consequences of eye movements, we remind readers that visual scenes are typically dynamic, with much fundamental information conveyed by movement (e.g., Johansson, 1973). Given that neither the world nor the eyes are typically stationary, the system has clearly evolved to deal with dynamic signals, whether generated by external motion, exploratory eye movements, blinks, or body motion through space. The recent work discussed here revealing the exquisite efficiency of the mechanisms attuned to the spatiotemporal signals generated by the various classes of eye movements, both large and small—the most common source of visual transients—should therefore come as no surprise.
